# Palmoplantar pustulosis: pathogenesis, differential diagnosis, and treatment

**DOI:** 10.1111/ddg.70238x

**Published:** 2026-04-08

**Authors:** Rotraut Mössner, Tanja Fetter, Robert Sabat, Ulrich Mrowietz, Neda Cramer, Dagmar Wilsmann‐Theis

**Affiliations:** ^1^ Department of Dermatology Venereology and Allergology University Medical Center Göttingen Göttingen Germany; ^2^ Center for Skin Diseases Department of Dermatology and Allergology University Hospital Bonn Bonn Germany; ^3^ Translational Skin Inflammation Research Department of Dermatology Venereology and Allergology Charité – Universitätsmedizin Berlin corporate member of Freie Universität Berlin and Humboldt‐Universität zu Berlin Berlin Germany; ^4^ Center for Inflammatory Skin Diseases Department of Dermatology Venereology and Allergology University Medical Center Schleswig‐Holstein Campus Kiel Kiel Germany

**Keywords:** palmoplantar pustulosis, pustulosis palmoplantar, palmoplantar pustular psoriasis, SAPHO, PAO, therapy

## Abstract

Palmoplantar pustulosis (PPP) is a chronic inflammatory and often painful disease characterized by sterile pustules on the palms and soles, significantly impairing quality of life. Women are more frequently affected than men, and smoking is a major trigger. Under biologic therapies, especially TNF antagonists, a paradoxical PPP may occur. PPP is associated with psoriasis vulgaris and may be accompanied by osteoarticular involvement. Pathogenetically, PPP likely begins around the acrosyringium, with the pustules consisting almost exclusively of infiltrating neutrophilic granulocytes attracted by chemotactic factors secreted by activated keratinocytes. Inflammation is sustained through a self‐amplifying cytokine network, including interleukin (IL)‐17, IL‐19, and related mediators.

Treatment options for PPP include topical treatments, UV‐phototherapies ‐ particularly topical PUVA (Psoralen plus UVA) therapy‐ and systemic therapies. Systemic agents comprise conventional treatments such as acitretin, methotrexate, fumaric acid esters, and ciclosporin, newer small molecules like apremilast and Janus kinase inhibitors, as well as biologics. Conventional systemic therapies are often not sufficiently effective in PPP and associated with side effects. Currently, among systemic therapies, only acitretin is approved for PPP. In recent years, placebo‐controlled studies have demonstrated a significant effect of apremilast, brodalumab, guselkumab and risankizumab on PPP, and further studies with topical and systemic Janus kinase inhibitors as well as IL‐17A/F inhibitors are underway.

## INTRODUCTION

Apart from psoriasis vulgaris (PV), there is also the so‐called pustular psoriasis. Historically, this comprises generalized forms, such as generalized pustular psoriasis (GPP) with its special forms, and localized forms occurring predominantly on hands and feet. The latter include palmoplantar pustulosis (PPP) as most common form described by Barber in 1930 as a pustular form of psoriasis,^1^ pustular bacterid (Andrews) described as acute PPP in 1934,^1^, ^2^ and acrodermatitis continua suppurativa of Hallopeau affecting the nail organ. Classification of these pustular diseases as special forms of psoriasis was already controversial at an early time. Originally, the poor therapeutic response of PPP compared to PV was noticed among other factors.^3^ Later, the view that pustular forms of psoriasis are independent entities was confirmed by genetic differences.^3^, ^4^ Accordingly, the term palmoplantar pustulosis (PPP) is increasingly used instead of the earlier term palmoplantar pustular psoriasis.^5^ In 2017, the *European Rare and Severe Expert Network* (ERASPEN) defined PPP as primary, persistent (> 3 months), sterile, macroscopically visible pustules of palms and/or soles.^6^

This article presents basic pathophysiological characteristics and current therapies of PPP.

Palmoplantar pustulosis is a chronic, recurrent skin disease characterized by sterile pustules on erythematous base on plantar and/or palmar areas associated with pruritus and pain.

## EPIDEMIOLOGY AND CLINICAL PRESENTATION

**FIGURE 1:**
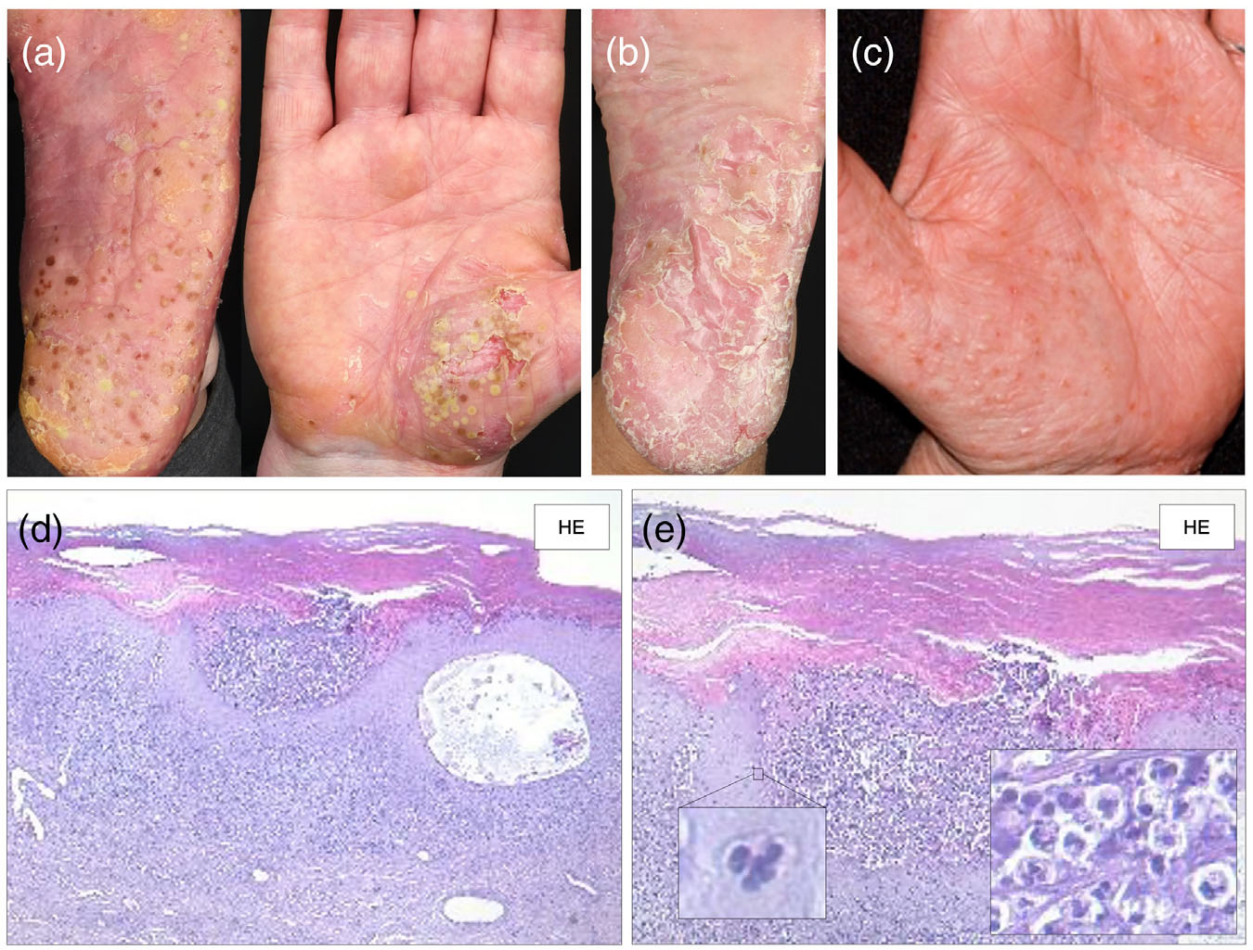
Clinical variants and histological features of palmoplantar pustulosis (PPP). The predilection sites of PPP are primarily the medial arch of the sole, as well as the lateral borders and the heels and on the palm the thenar (a) and hypothenar regions. The sterile pustules may coalesce and, over time, resolve as brown macules (a) and/or by forming hyperkeratotic plaques resembling plaque psoriasis (b). (a) Pustular variant. (b) Hyperkeratotic variant, (c) Dyshidrosiform variant, (d) and (e) Histology of PPP. Palmoplantar pustulosis with Kogoj macropustules, consisting of dense intraepidermal accumulations of neutrophilic granulocytes. Munro microabscesses may occasionally be present in the stratum corneum. The dermis shows a mixed‐cell inflammatory infiltrate with dilated capillaries; eosinophils and mast cells can be found beneath the pustules.

PPP is a chronic or chronic recurrent disease manifesting clinically with pustules, often on an erythematous, sometimes scaly base on palms and/or soles.^7^ (Figure 1). For the plantar aspect, the medial arch of the sole, as well as the lateral borders and the heels are primarily affected; on the palms, the changes occur primarily in the thenar and hypothenar region. The sterile pustules may coalesce and resolve over time as brown macules and/or under formation of hyperkeratotic plaques resembling plaque psoriasis^8^, ^9^ In addition, the clinical spectrum of PPP includes vesicles that may progress to pustules^10^ (Figure 1). 30–76% of the patients with PPP present with nail changes, especially onycholysis through to nail destruction.^8^, ^11^ PPP may be present for decades with phases of partial or complete remission followed by exacerbations.^12^, ^13^, ^14^ The skin lesions are often associated with severe pain and pruritus.^12^

**FIGURE 2:**
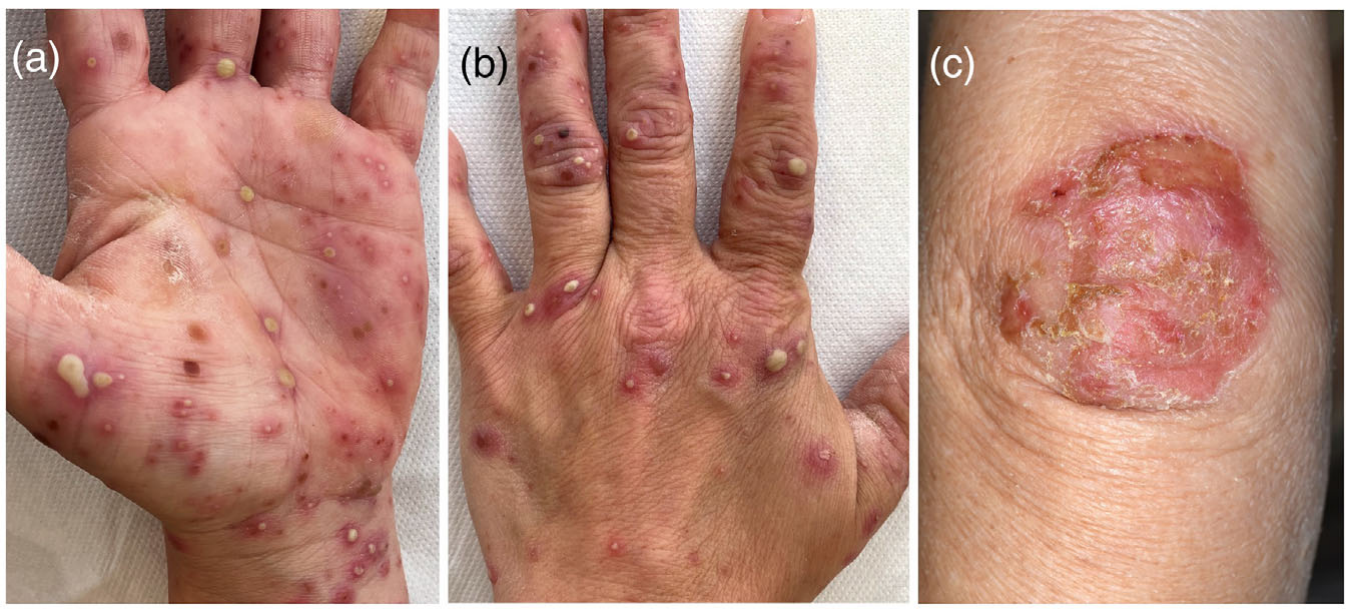
Typical clinical presentation of acute PPP on the palm with large pustules with a red peripheral halo (a), involvement of the dorsum of the hand with isolated pustuleswith a red peripheral halo (b), as well as hyperkeratotic‐erosive plaques on the elbow (c) on otherwise unaffected skin.

From this (chronic) PPP, an acute PPP is distinguished that is also referred to as pustular bacterid (Andrews),^1^ (Figure 2). This is often preceded by bacterial infection, often caused by streptococci and primarily affecting the tonsils.^5^ It was originally described as self‐limiting pustular id reaction, but this remains controversial. The clinical presentation is usually characterized by isolated, often larger pustules between 5 and 10 mm in diameter with red peripheral halo on otherwise unaffected skin. While the pustules affect primarily the palmoplantar regions, they may also occur on the dorsal aspects of hands and feet and occasionally on the remaining skin (Figure 2).^5^, ^15^

The prevalence of PPP is not known precisely. In Germany, a prevalence of 0.065% was obtained by means of the IQVIA *Disease Analyzer Database* using ICD‐10‐CM L40.3, while a prevalence of 0.005% was found in the USA (obtained by means of the *US MerativeTM MarketScan Commercial Database*).^16^ In Japan, it seems to be higher than in Western countries (up to 0.12%).^16^, ^17^, ^18^, ^19^ With a proportion between 58% and 94%, women are more often affected by PPP than men.^8^ The disease severity appears to be higher in women than in men.^17^ In a German study, patients developed the disease most often at an age from 40 to 59 years, with a mean of 41.6 years.^20^ PPP is associated with a severely reduced quality of life.^20^ Based on measurements with the *Palmoplantar Pustulosis Area and Severity Index* (PPPASI) and *Dermatology Life Quality Index* (DLQI), there is a positive correlation between severity of PPP and impairment of the health‐related quality of life.^14^, ^15^, ^16^, ^17^, ^18^, ^19^, ^20^

The severity of PPP is evaluated with both patient‐oriented scores, such as DLQI and evaluation of pain and pruritus (visual analog scale, numeric rating scale), and objective diagnostic scores, such as PPPASI^21^ or/and the less time‐consuming PPP‐PGA (*Physician Global Assessment*) (Table 1; Table 2).^22^

In Germany, the prevalence of PPP has been estimated at 0.065%, and postmenopausal women are predominantly affected. Smoking tobacco is a significant trigger factor.

**TABLE 1:** Score for assessing the objective severity of palmoplantar pustulosis (PPP): PPP‐Physician Global Assessment (PGA);

PGA scale	Short description	Detailed description	Clinical example
0	clear	No signs of PPP, no scaling or crusts or remains of pustules	
1	almost clear	Minimal scaling and/or minimal erythema and/or mild crusts, very few new (yellow) and/or old (brown) pustules	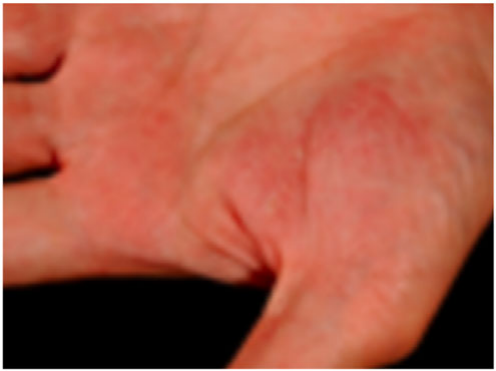
2	mild	Mild scaling and/or mild erythema and/or crusts; visible new (yellow) and/or old (brown) pustules of limited number and extension	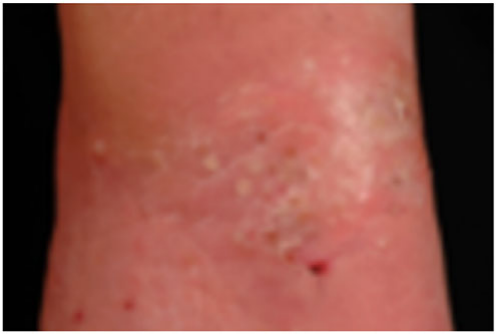
3	moderate	Moderate scaling and/or pronounced erythema and/or crust formation; conspicuous new (yellow) and/or old (brown) pustules, covering the major part of the affected area	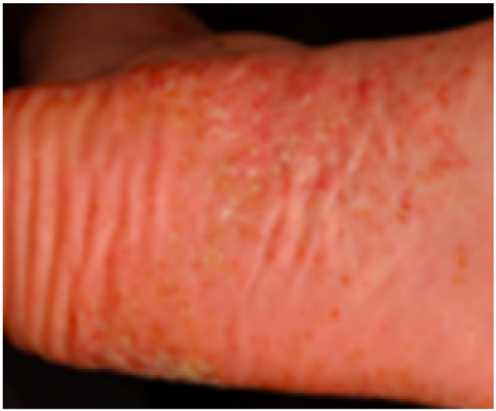
4	severe	Severe scaling and/or severe erythema and/or crust formation; numerous new (yellow) or old (brown) pustules with and/or without coalescence covering the entire area of at least 2 palmoplantar surfaces	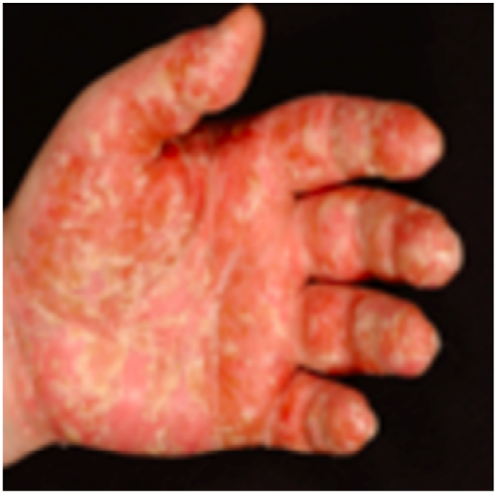

**TABLE 2:** Score for assessing the objective severity of palmoplantar pustulosis (PPP) Palmoplantar Pustulosis Area and Severity Index (PPPASI).

Body site	Erythema (E)	Pustules/Vesicles (P)	Scaling (Desquamation) (D)	Extent of involvement (A)
**Right palmar** **(RP)**	Score 0–4	Score 0–4	Score 0–4	Score 0–6
**Left palmar** **(LP)**	Score 0–4	Score 0–4	Score 0–4	Score 0–6
**Right sole** **(RS)**	Score 0–4	Score 0–4	Score 0–4	Score 0–6
**Left sole** **(LS)**	Score 0–4	Score 0–4	Score 0–4	Score 0–6
**Formula for** **calculation**	**PPPASI** = 0.2 x (ERP + PRP + DRP) x ARP + 0.2 x (ELP + PLP + DLP) x ALP + 0.3 x (ERS + PRS + DRS) x ARS + 0.3 x (ELS + PLS + DLS) x ALS			
Discussion Score according to involvement	0 = no involvement 1 = mild 2 = moderate 3 = severe 4 = very severe	0 = no involvement 1 = mild 2 = moderate 3 = severe 4 = very severe	0 = no involvement 1 = mild 2 = moderate 3 = severe 4 = very severe	0 = no involvement < 10% = 1 10–29% = 2 30–49% = 3 50–69% = 4 70–89% = 5 90–100% = 6
Total score	72 (maximum)			

### Trigger factors

One important trigger factor is smoking tobacco. In a German collective, 95% of the patients with PPP were smokers or former smokers.^20^ Moreover, PPP was more severe in smokers compared to former smokers and non‐smokers,^23^ and in a small study PPP improved after cessation of smoking.^24^ Furthermore, associations of PPP with infections, especially tonsillitis or periodontitis, have been particularly described in Japanese patients.^25^ In a retrospective Japanese study, 87% of the 85 included patients had an untreated dental infection.^26^ In this collective, a marked improvement of PPP was documented in 39% of the patients after successful dental treatment. In five of six patients with tonsillitis, PPP markedly improved after tonsillectomy.^26^ In addition, there are also larger studies, predominantly conducted in Japan, on the therapeutic effect of tonsillectomy in refractory PPP patients. In a retrospective Japanese study on 138 patients, 43% were free of signs and symptoms at their final consultation, and in a Kaplan‐Meier analysis, PPP had disappeared in 38% and 66% of the patients within twelve and 24 months, respectively.^25^ Interestingly, neither the aggravation of PPP during throat infections nor the presence of tonsillar hypertrophy were clinical markers for an improvement of PPP after tonsillectomy.^25^

Mechanical or chemical irritations, wet work, and heat may also result in aggravation of PPP.^17^, ^27^, ^28^, ^29^ Given that PPP often results in long‐term sick leave, it should be verified whether PPP is significantly affected by the occupation and should be reported to the relevant workers’ compensation association. If it becomes apparent that the skin manifestations are caused or aggravated by performing an activity hazardous to the skin in the sense of BK No 5101, the relevant accident insurer should be notified, in Germany by using a dermatologist's report (initiation dermatologist's report, form F6050). More often, however, the occupational effects in PPP will not exceed the extent of a so‐called occasional cause.^29^

Patients with PPP report psychological stress as a trigger or cause of aggravation.^20^ Contact allergies as trigger factors of PPP are controversially discussed. A systematic review revealed epicutaneous sensitizations, especially to metals, in 22.7% of PPP patients.^30^ In this review, improvement of PPP in patients with positive patch test was observed after removing dental fillings or dentures,^30^ although in one study such an improvement was attributed more to the simultaneous treatment of dental infections,^31^ and in another study, six of nine patients showed no improvement after removal of metals from the oral cavity.^26^

Paradoxical PPP may manifest primarily as a  side effect of TNF inhibitors, particularly in patients with inflammatory bowel disease.

### Paradoxical PPP

Under treatment with certain biologics, psoriatic skin lesions have been observed as so‐called paradoxical reactions. In this context, the term paradoxical is used to indicate that the induced side effect resembles a disease that is treated by the triggering medication.^5^ The paradoxical psoriasiform reactions may manifest as aggravation of an existing psoriasis, change of the clinical presentation (for example, the first appearance of a pustular variant), or the first manifestation of psoriasiform skin lesions.^5^ 14% to 75% of the paradoxical psoriasiform reactions are pustular. In most cases the palms and soles are affected, which is also referred to as paradoxical PPP, while a generalized involvement has been observed in about 22% of the patients with a pustular variant.^32^ Most often, paradoxical PPP occurs in therapies with tumor necrosis factor (TNF)‐α inhibitors.^33^ In rare cases, paradoxical PPP was also described for interleukin (IL)‐17 or IL‐17RA antagonists.^34^ The latency after initiation of treatment with a TNF inhibitor is, on average, 10.5 months and ranged from several days to 80 months.^35^ While paradoxical psoriasiform reactions induced by TNF inhibitors were observed in all approved indications, they were particularly common in patients with inflammatory bowel disease and less common in patients with rheumatoid arthritis.^5^ In a meta‐analysis on patients with inflammatory bowel disease, female gender, smoking, younger age at initiation of anti‐TNF therapy, patients with ileocolic type of Crohn's disease, and the type of TNF inhibitor (adalimumab and certolizumab versus infliximab) were identified as risk factors for paradoxical psoriasiform reactions.^36^ It is hypothesized that inhibition of TNF results in overactivation of the type I interferon (IFN) signaling pathway, which contributes significantly to the formation of skin lesions.^37^

## ASSOCIATED DISEASES

PPP is frequently associated with PV.^38^ In a German collective, 25% of the patients with PPP had also PV and 50% of those reported that PV manifested in the same year as PPP.^20^ In that study, 30.2% of the patients with PPP had a positive family history of psoriasis.^20^ In studies on the gene expression of lesional skin of patients with PPP, no significant differences were found between the subgroups with or without lesions typical of psoriasis on the remaining skin. Therefore, it remains unclear whether pathogenetic differences exist between PPP with concomitant PV and PPP without PV.^39^

### Bone and joint involvement

PPP is associated with bone and joint involvement with diverse clinical presentation. Central features are an involvement with aseptic osteitis with osteomyelitis presenting as bone marrow edema in magnetic resonance imaging and hyperostosis with periostitis ossificans.^40^, ^41^ A typical example is an inflammation of the anterior thoracic chest wall manifesting clinically with swelling and tenderness of the manubriosternal or costoclavicular regions. In Japan, Sonozaki has coined the term pustular arthro‐osteitis for this manifestation.^42^, ^43^ The definition of pustular arthro‐osteitis, for which the diagnosis of PPP is mandatory, was later modified with respect to the osteoarticular criteria that now include inflammatory involvement of the spinal column, sacroiliac joints, or other bones.^44^ In Japan, the prevalence of pustular arthro‐osteitis in patients with PPP is 10–30%.^41^ In Germany, the term pustular arthro‐osteitis is less well‐known, more familiar is the Synovitis, Acne, Pustulosis, Hyperostosis, Osteitis (SAPHO) syndrome originally defined in 1987 based on a national survey in France.^45^ Four main criteria were specified there and each criterion is sufficient for diagnosing SAPHO syndrome, provided that no exclusion criteria (like infectious osteitis) exist. Two of the four main criteria are associated with pustular skin diseases: 1) osteoarticular involvement if severe acne is present and 2) osteoarticular involvement if PPP is present. The two other main criteria may be met without skin disease: they are 3) sterile hyperostosis (hyperostosis is considered sterile even if *Cutibacterium acnes* (previously *Propionibacterium acnes*) is detected) with or without dermatosis and 4) chronic recurrent multifocal osteomyelitis (CRMO) of the axial or appendicular skeleton with or without dermatosis. In this context, SAPHO syndrome is understood as a symptom complex and not as an individual disease entity, and modifications of the criteria of SAPHO syndrome have been proposed (Table 3).^40^ A characteristic diagnosis in this symptom complex is, for example, pustular‐psoriatic hyperostotic spondyloarthritis, the mandatory triad of palmoplantar pustulosis, sternocostoclavicular hyperostosis, and (hyperostotic) spondyloarthritis. The SAPHO syndrome also includes pustular arthro‐osteitis.^40^, ^41^, ^46^ At the time when PPP was mostly not classified as an individual entity, but rather as belonging to the psoriatic disease spectrum, bone and joint involvement were also understood as psoriatic arthritis (PsA).^47^ In a study conducted in South Korea, patients with pustular arthro‐osteitis and PsA were compared. There too, based on differences in demographic data and the affected joints, pustular arthro‐osteitis and PsA are understood as distinct entities.^48^ Additional studies on the question whether bone and joint involvement in patients with PPP differ in populations with different genetic backgrounds are desirable.

**TABLE 3:** Tabular overview of diagnostic criteria for pustular arthro‐osteitis (Sonozaki syndrome), SAPHO syndrome, and psoriatic arthritis to differentiate musculoskeletal manifestations in palmoplantar pustulosis (PPP).

**Pustular arthro‐osteitis = Sonozaki‐Syndrom**	
**Diagnostic criteria^a^ according to Sonozaki et al. (1981)** ^42^ **(adapted from Kishimoto et al. (2022)** ^41^	
1) Diagnosed palmoplantar pustulosis	
2) Patients meeting one of the two following criteria: apparent swelling tender to palpation on either side of the costoclavicular or manubriosternal regions, with or without positive x‐ray findings tenderness without apparent swelling on either side of the costoclavicular or manubriosternal regions, with positive x‐ray finding at the site tender to palpation	
*Diagnosis is made when the following is met:* *Criterion 1) and criterion 2)*.	
*^a^* The diagnostic criteria were modified later, see for example *Tsuji et al. (2024)* ^44^, *Nagel et al. (1993)* ^43^	
**SAPHO‐Syndrom**	
**Diagnostic criteria adapted from Kishimoto et al. (2022)** ^41^ **according to Hayem et al. (2004)** ^46^	
1) Inclusion criteria: Bone‐joint involvement associated with PPP and psoriasis vulgaris Bone‐joint involvement associated with severe acne Isolated sterile hyperostosis/osteitis (adults) Chronic recurrent multifocal osteomyelitis (children) Bone‐joint involvement associated with chronic inflammatory bowel diseases	
2) Exclusion criteria: Infectious osteitis Tumoral condition of the bone Non‐inflammatory condensing lesions of the bone	
*Diagnosis is made if the following is met:* *One inclusion criterion, no exclusion criterion*.	
**Psoriatic arthritis**	
**CASPAR classification criteria according to Taylor et al. (2006)** ^50^	
*Diagnosis is made if the following is met:* *Inflammatory condition of joints, spinal column, and tendons/entheses with at least 3 items from the following 5 categories*:	
**Evidence of Psoriasis**	
Current psoriasis	2 oder
Personal history of psoriasis (information provided by patient, family doctor, dermatologist, or rheumatologist)	1 oder
Family history of psoriasis (known history of psoriasis in a first‐degree/second‐degree relative according to patient report)	1
**Typical psoriatic nail dystrophy**	
Pitting, onycholysis, hyperkeratosis on current physical examination	1
**Negative test result for presence of rheumatoid factor**	
Preferably by ELISA or nephelometry, according to the local test reference range	1
**Dactylitis**	
Current dactylitis (swelling of the entire digit)	1 oder
History of dactylitis (diagnosis by rheumatologist)	1
**Radiologic signs of juxta‐articular formation of new bone**	
Ill‐defined ossification near the joint margins (but excluding osteophyte formation) on x‐ray images of hands or feet	1

Abbr.: CASPAR = Classification Criteria for Psoriatic Arthritis; ELISA = Enzyme‐Linked Immunosorbent Assay; SAPHO = Synovitis, Acne, Pustulosis, Hyperostosis, Osteitis

PsA is a chronic inflammatory disease of the musculoskeletal system with arthritis, enthesitis, spondylitis, and/or dactylitis.^49^ The Classification for Psoriatic Arthritis (CASPAR) criteria are used as diagnostic criteria for PsA (Table 3).^50^ Psoriasis (for example, plaque psoriasis) is assigned a score of 2, and the CASPAR criteria are then usually met (after exclusion of differential diagnoses) if inflammatory joint disease is present (for example, the additional presence of rheumatoid factor negativity is sufficient). Given that PPP is increasingly no longer considered a subtype of psoriasis, the CASPAR criteria can only be met in case of bone and joint involvement if additional criteria, such as positive family history of psoriasis or psoriatic nail changes, are present. In Germany, the prevalence of joint involvement in patients with PPP was 16–28% (at that time assessed as PsA, given that PPP was still considered a subtype of psoriasis).^14^, ^20^

Approximately 25% of the patients with PPP have also psoriasis vulgaris. Osteoarticular involvement is present in 16–28% with diverse manifestations; one typical manifestation is aseptic osteitis/hyperostosis.

### Additional associations

Approximately 30% of the patients with PPP had metabolic syndrome. In studies conducted in Germany, approximately a fourth of the patients with PPP had a BMI > 30.^14^, ^20^ With a value of 27.7, the BMI was strongly increased in particular in younger patients with PPP, while in the age group of 65 years and above, the BMI of patients with PPP was 24.3 and thus lower than in the general population.^20^ In PPP, a higher prevalence of thyroid dysfunction and elevated serum levels of anti‐thyroid peroxidase have been observed, while in a study from South Korea, Graves’ disease was more prevalent in patients with PPP than in patients with PV.^51^, ^52^ Although the presence of other cardiovascular, metabolic, and autoimmune diseases in patients with PPP has been described, the strength of the association with PPP compared to the general population has not yet been clarified. In addition, a higher prevalence of psychiatric disorders, such as depression, bipolar disorder, schizophrenia, as well as anxiety and eating disorders has been identified in patients with PPP.^14^, ^53^, ^54^

## GENETICS

PPP differs genetically from PV. In particular, PPP is not associated with the *PSORS1* locus (HLA‐Cw* 06:02), the most important susceptibility locus of PV.^55^ There is also no association of PPP with mutations in the *IL36RN* gene, which is associated with generalized pustular psoriasis.^56^

In a genome‐wide meta‐analysis, a significant association of PPP with the FCGR3A/FCGR3B loci encoding receptors of immunoglobulins and the CCHCR1 locus were identified. In addition, 13 other likely susceptibility regions for PPP were found.^57^ Overall, immunological signaling pathways in the skin and of circulating dendritic cells were particularly affected. Correlations were also found with *single nucleotide polymorphisms* (SNPs) in the IL‐4/IL‐13 gene region encoding Th2 cytokines. In line with these findings, a genetic correlation analysis showed a correlation with atopic dermatitis but not PV and a negative correlation with ulcerative colitis.^57^

The inflammation of PPP is sustained by a self‐amplifying mechanism involving IL‐17, IL‐19, and additional mediators further stimulating neutrophil chemotaxis.

## PATHOGENESIS

The pathogenesis of PPP is not yet fully understood. The disease appears to begin around the acrosyringium, the intraepidermal secretory duct of the eccrine sweat glands,^10^, ^58^ while the pustules consist almost exclusively of infiltrating neutrophilic granulocytes. These neutrophils are attracted by chemotactic factors (CXCL8, lipocalin‐2) secreted by activated keratinocytes. In vitro studies indicate that nicotine acts as a trigger factor amplifying this process.^59^, ^60^

The emerging pustules consist almost exclusively of infiltrated neutrophilic granulocytes. In line with a central role of these cells in the pathogenesis of PPP are recent results of a genome‐wide association meta‐analysis that identified an association of PPP with the gene loci of receptors for immunoglobulins (FCGR3A/FCGR3B).^57^ FCGR3B is indeed selectively expressed by neutrophilic granulocytes and eosinophils, and as *decoy* receptor it can modulate inflammatory processes in various ways. Apart from the chemokine ligand CXCL6,^61^, ^62^ additional chemotactic factors, such as CXCL1, CXCL8, and lipocalin‐2, may play a role in the infiltration of these cells into the epidermis.^59^, ^63^ These proteins are secreted by activated keratinocytes. However, it is currently unclear what causes the keratinocytes of the acrosyringium to produce these chemotactic factors. Possible inducers are immune mediators like IL‐1β and TNF^59^ produced by tissue‐resident immune cells, such as dendritic cells and macrophages, and components of sweat. Nicotine as a component of tobacco smoke, the most important etiological factor of PPP, amplifies the chemotaxis of neutrophilic granulocytes.^60^

**FIGURE 3:**
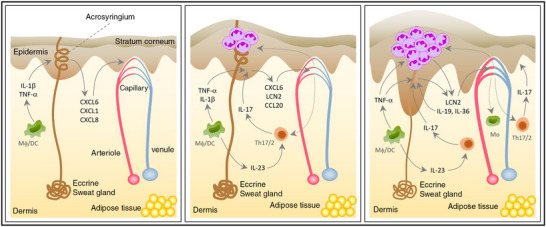
Pathogenesis of Palmoplantar Pustulosis (PPP). The schematic illustration shows the current understanding of PPP pathogenesis in three consecutive stages. (a) The disease begins around the acrosyringium. In this area of the epidermis, keratinocytes release chemotactic factors (C‐X‐C Motif Chemokine Ligand (CXCL)1, CXCL6, CXCL8) that attract neutrophilic granulocytes. Possible activators causing keratinocytes to do this include sweat components and inflammatory cytokines: interleukin (IL)‐1β and tumor necrosis factor (TNF)‐α produced by activated tissue‐resident immune cells (macrophages, dendritic cells; Mf/DC). (b) Under the influence of IL‐23, T helper (Th) 17/Th2 cells are activated, secreting IL‐17. This intensifies the production of chemotactic mediators (including CXCL6, lipocalin‐2 [LCN2], and CCL20) by keratinocytes. Neutrophilic granulocytes accumulate intraepidermally to form the typical Kogoj macropustules of PPP. (c) The infiltrated neutrophilic granulocytes produce IL‐19 and LCN2. IL‐19 enhances the effect of IL‐17 on keratinocytes, similar to IL‐1β and TNF‐α, and LCN2 promotes further infiltration and activation of neutrophilic granulocytes in the skin. The inflammatory cycle becomes chronic and self‐perpetuating.

In PPP lesions, the production of proteins from keratinocytes attracting neutrophilic granulocytes into the epidermis may be further amplified by mediators like IL‐17 and IL‐19.^61^, ^64^ IL‐17A is produced by infiltrated T cells and IL‐19 by neutrophilic granulocytes.^61^, ^64^, ^65^ The infiltrated T cells are predominantly found in the upper dermis^28^ and appear to have a Th17/Th2 transition phenotype.^66^ These T cells also express the IL‐23 receptor, and IL‐23 can have a positive effect on their activity.^65^, ^66^ Apart from IL‐19, neutrophilic granulocytes infiltrated into the epidermis also express lipocalin‐2, which acts as positive amplifier attracting neutrophilic granulocytes into the epidermis.^59^, ^67^ The effect of IL‐17 on keratinocytes, the main target cells of this cytokine, is amplified by IL‐1β and TNF (Figure 3).^59^, ^65^ The concentrations of both lipocalin‐2 and IL‐19 are elevated in the blood of patients with PPP, and the levels of these mediators correlate with the number of pustules.^59^, ^61^

## HISTOLOGY OF PPP

**FIGURE 4:**
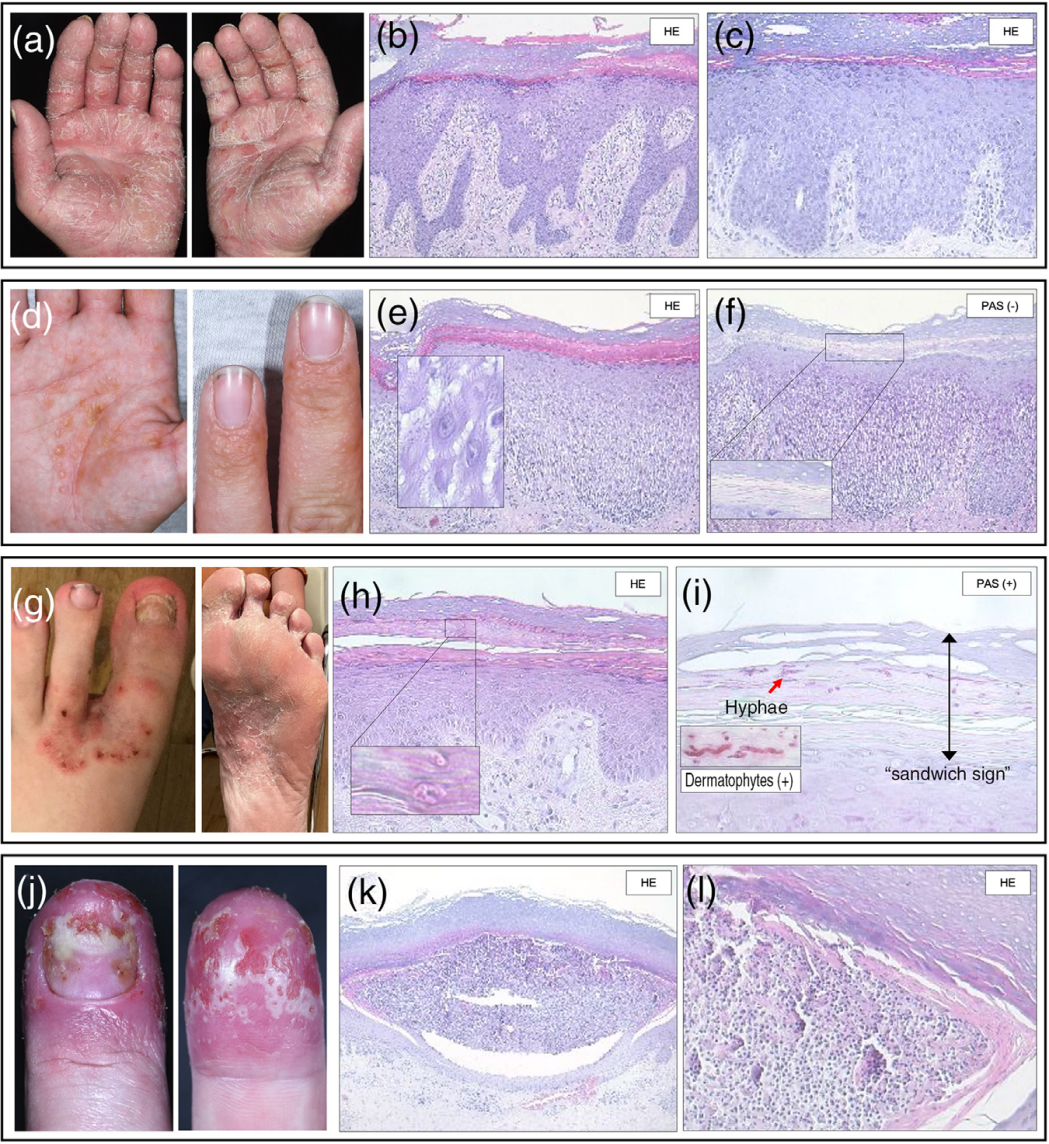
Clinical and histological differential diagnoses of palmoplantar pustulosis (PPP). (a–c) Chronic hand eczema with hyperkeratosis, parakeratosis, acanthosis, and a perivascular lymphocytic infiltrate, but without intraepidermal pustules. (d–f) Dyshidrosiform eczema with pronounced epidermal spongiosis and vesicle formation, but without neutrophilic infiltrates. Periodic Acid‐Schiff (PAS) staining remains negative. (g–i) Tinea with PAS‐positive fungal hyphae in the stratum corneum (‘sandwich sign’). These findings are crucial for distinguishing PPP, especially in vesicular or pustular lesions on palms or soles. PAS staining is therefore always recommended in such cases. (j–l) Acrodermatitis continua suppurativa (Hallopeau) with similar intraepidermal, spongiotic pustules filled with neutrophilic granulocytes, typically localized and often associated with nail changes. The pustules are accompanied in the superficial and mid‐dermis by a perivascular and interstitial lymphocytic infiltrate.

Histologically, the presence of so‐called Kogoj macropustules presenting the intraepidermal accumulations of neutrophilic granulocytes is characteristic for PPP. Occasionally, so‐called Munro microabscesses, which also present accumulations of neutrophils and are a typical feature of PV, may be observed in the stratum corneum (Figure 4). Accumulation of eosinophils and mast cells may be observed below the pustule.^28^, ^58^ The dermis presents with a mixed‐cell lymphocytic inflammation, often associated with dilated capillaries. Other histological features characteristic for PV, such as loss of stratum granulosum, elongated rete ridges/acanthosis, and hyperkeratosis with parakeratosis, are not always present and tend to occur more frequently in older and persistent lesions.^2^, ^28^, ^58^, ^68^ In early stages of PPP, spongiosis and vesicle formation, which may resemble pompholyx in histology, are occasionally observed.^2^, ^58^ If vesicular and pustular lesions are present on palms and soles, PAS staining should always be performed, especially if neutrophilic granulocytes are present in vesicles or stratum corneum, given that dermatophyte infections may mimic and/or accompany PPP and pompholyx lesions.^69^, ^70^

Histologically, PPP is primarily characterized by accumulation of neutrophilic granulocytes within the epidermis, the so‐called Kogoj macropustules. In early stages, the picture may resemble pompholyx (dyshidrosiform eczema) due to intraepidermal spongiosis and vesicle formation.

## DIFFERENTIAL DIAGNOSIS

Differential diagnoses of PPP include acute PPP, palmoplantar psoriasis (without pustules), acrodermatitis continua of Hallopeau, dyshidrosiform eczema with pompholyx as maximum variant, chronic hand eczema, and tinea manuum or pedis (Figure 4).^71^

## THERAPY OF PPP

PPP is a chronic disease requiring long‐term treatment and often combinations of topical and systemic therapies. Given the lack of specific guidelines and randomized trials, the therapy is often based on that for PV.^72^ In contrast to PPP, the paradoxical PPP has a better prognosis. In a recently published meta‐analysis of 155 patients with drug‐induced PPP, 58.8% of the patients showed complete remission after approximately four months, especially on topical corticosteroids or after switching the biologic agent.^73^ Given that the co‐existing underlying inflammatory disease also requires treatment, the selection of a systemic therapy in “paradoxical PPP” should be made in an interdisciplinary manner.

Topical therapies of PPP include ultrapotent corticosteroids under occlusion or the fixed‐dose combination of calcipotriol and betamethasone *(off label)*, while Janus kinase inhibitors (JAKi) might present a further  *off label* option.

### Topical therapy

While topical treatments are often the first approach in PPP, they reach their limits due to the low penetration into the stratum corneum of soles and palms and the chronicity of PPP. Although the penetration of ultrapotent topical corticosteroids may be improved by occlusion, this is only feasible in the short term.^47^, ^74^, ^75^ While vitamin D3 analogs like maxacalcitol were effective especially in combination with betamethasone in Japanese patients with PPP, this drug is not approved for treatment of PPP in Germany.^76^, ^77^ However, the fixed‐dose combination of betamethasone and calcipotriol, preferably as aerosol foam and as cream based on *polyaphron dispersion* (PAD) technology, may be used.^78^, ^79^ Janus kinase inhibitors (JAKi) might present a further option for topical treatment of PPP; so far, one promising case of PPP treated with ruxolitinib has been published.^80^

Phototherapies are time‐ and resource‐intensive. They are often used for bridging until the onset of action of a newly initiated systemic therapy.

### Ultraviolet (UV) radiation

UV radiation represents a supportive treatment option. Based in the potential acute and chronic long‐term risks of photochemotherapy, the indication in chronic recurrent skin diseases requiring repeated radiation cycles should be somewhat stricter.^81^

In Japanese patients, narrow‐band UVB phototherapy showed a significant improvement of PPPASI by 61.4% after twelve weeks.^82^ Standard treatment is, however, PUVA photochemotherapy (psoralen plus UVA radiation), which is approved as bath PUVA for PPP.^83^ Studies indicate a slight advantage of UVA/PUVA over UVB radiation.^51^, ^72^, ^81^, ^84^ In clinical practice, a very good therapeutic response of topical PUVA may be observed in some cases. However, this has not always been verified in prospective studies, which also used shorter exposure times (ten minutes).^85^ The combination of PUVA with retinoids (Re‐PUVA) may achieve synergistic effects.^86^

Excimer laser therapy (xenon chloride (XeCl) laser with a wavelength of 308 nm) presents another therapeutic option.^87^ In a Chinese prospective comparative study with 73 patients, the excimer laser showed a PPPASI‐75 response of 95% in the high‐dose group compared to 8.3% and 29.17% in the low‐dose group and medium‐dose group, respectively, after twelve weeks with three applications per week.^87^, ^88^, ^89^ Doses of at least 200 mJ/cm^2^ 1 to 2 times a week and 10 to 20 excimer settings have been reported.^90^, ^91^ Especially for the milder form of PPP induced by TNF‐α, where the existing systemic therapy should be continued due to the otherwise positive effect, this might represent a good alternative treatment option.^33^, ^35^

## SYSTEMIC THERAPY

Of the systemic therapies used in PPP, only acitretin is approved for treatment.^92^ Experience concerning other therapies is derived from clinical studies, observations from the therapeutic effect of systemic therapies administered *in label* for an accompanying disease (for example, plaque psoriasis, psoriatic arthritis, seronegative spondyloarthropathy, or inflammatory bowel disease), and from treatments used *off label*.

Acitretin is the only approved systemic therapy of PPP in Germany; however, its use is limited due to comorbidity, such as hyperlipidemia usually in the context of the metabolic syndrome, and because it is teratogenic. The other conventional systemic therapeutics also have limitations, although ciclosporin A may be quite effective.

### Retinoids

Acitretin is the only drug explicitly approved for PPP.^92^ Two prospective studies with acitretin and etretinate, respectively, showed a significant reduction in clinical findings in patients with PPP after twelve weeks.^51^, ^93^, ^94^ A small placebo‐controlled study with alitretinoin, which is approved for chronic hand eczema, showed no superiority over the placebo group in patients with PPP.^95^ Important side effects of retinoids are dryness of skin and mucous membranes, hyperlipidemia, and potentially the increase of existing symptoms of depression.^92^

Acitretin is teratogenic. Its use is contraindicated in women who may become pregnant during treatment or within a period of three years after termination of treatment, unless all conditions of the contraception program are met (see corresponding summary of product characteristics).^92^

### Methotrexate (MTX)

The data available on MTX for the treatment of PPP in prospective studies or case series is insufficient.^96^ One retrospective analysis with 42 MTX and a total of 201 treatment courses showed a mean duration of administration of eight months that was extended to twelve months in combination with biologics. MTX is also considered in combination with biologics or if psoriatic arthritis is present as comorbidity.^97^

### Ciclosporin A (CsA)

Two randomized controlled trials (RCTs) with CsA in patients with PPP showed a significant reduction of the number of pustules after one month compared to placebo,^98^, ^99^ (Table 4). A prospective single‐arm study with CsA on 48 patients at a dosage of 3 mg/kg BW showed clinical improvement in 45 patients within 15–30 days (a primary endpoint was not defined).^100^ CsA is characterized by a fast onset of action, but long‐term treatment is limited due to side effects, such as arterial hypertension and nephrotoxicity.^101^ A German analysis showed an excellent response rate of 51.4% for CsA compared to 19.5% for acitretin and 16.8% for MTX and a longer *drug survival* of twelve months.^97^

**TABLE 4:** Overview of prospective studies with *small molecules* and biologics as treatment options for palmoplantar pustulosis (PPP). *Abbr*.: s.c. = subcutaneous; n.g. = not given; bid = twice daily; W = week; *primary endpoint achieved; +not placebo‐controlled.

Drug	Number of patients in verum group	Studied drug Clinical response rate Week 12–20		Placebo Clinical response rate Week 12–20		Reference
		PPPASI‐50 (%)	PPPASI‐75 (%)	PPPASI‐50 (%)	PPPASI‐75 (%)	
**Prospective placebo‐controlled trials achieving their primary endpoint**						
Brodalumab 210 mg s.c.	63	54.0	36.0	14.1	8.1	(Okubo et al. 2024)^133^
Guselkumab 100 mg s.c.	54	57.4	20.4	34.0	3.8	(Terui et al. 2019)^126^
Guselkumab 200 mg s.c.	52	36.5	11.5			
Guselkumab 200 mg s.c.	25	60	n.g.	21	n.g.	(Terui et al. 2018)^125^
Risankizumab 150 mg s.c.	61	41	13.1	24.1	15.5	(Okubo et al. 2025)^128^
Apremilast 30 mg bid	46	78.3	43.5	40.9	15.9	(Terui et al. 2023)^104^
Ciclosporin 2.5 mg/kg/day*	19	n.g.	n.g.	n.g.	n.g.	(Reitamo 1993)^98^
Ciclosporin 1 mg/kg/day*	27	n.g.	n.g.	n.g.	n.g.	(Erkko et al. 1998)^99^
**Prospective placebo‐controlled *trials NOT achieving their primary endpoint***						
Anakinra 100 mg s.c./day	31	21 W8	0 W8	16 W8	3 W8	(Cro et al. 2021)^137^
Alitretinoin 30 mg/day	24	50	23	67	33	(Reich et al. 2016)^95^
Secukinumab 150 mg s.c.	80	36.5	17.5	34.0	14.1	(Mrowietz et al. 2019 und 2021)^130^, ^131^
Secukinumab 300 mg s.c.	79	52.2	26.6			
Spesolimab s.c. Several dosing regimens	109	n.g.	n.g.	n.g.	n.g.	(Burden et al. 2023)^22^
Ustekinumab 45 mg s.c.	15	13.3	n.g.	37.5	n.g.	(Bissonnette et al. 2014)^63^
**Prospective placebo‐controlled *trials with significant effect but without clearly defined primary endpoint***						
Etanercept 50 mg s.c.	10	n.g.	n.g.	n.g.	n.g.	(Bissonnette et al. 2008)^119^

Clinical prospective single‐arm trials on palmoplantar pustulosis achieving their primary endpoint*				
**Drug**	**Number of patients**	**PPPASI‐50 (%)**	**PPPASI‐75 (%)**	**Reference**
Guselkumab 100 mg s.c.	50	66 (W24)	34 (W24)	(Wilsmann‐Theis et al. 2025)^127^
Apremilast 30 mg bid	20	61.9 (W20)	14.3 (W20)	(Wilsmann‐Theis et al. 2021)^103^
bid: twice daily; PPPASI: Palmoplantar Pustulosis Area and Severity Index; s.c. subcutaneous; W: Week *Only trials with more than 12 patients were included				

*Abbr*.: s.c. = subcutaneous; n.g. = not given; bid = twice daily; W = week; *primary endpoint was achieved; +not placebo‐controlled

### Fumaric acid esters

In a clinical study on fumaric acid esters with 13 patients, eight patients achieved a reduction of the clinical score for PPP by 49% on the hands and 44% on the feet at week 24.^102^ Gastrointestinal symptoms and flush are considered common limiting side effects, and the delayed onset of action further limits the use of fumaric acid esters for treatment.

Modern *small molecules*, such as apremilast and Janus kinase inhibitors, might present themselves as more effective in PPP than in PV.

### New small molecules

#### Apremilast, phosphodiesterase (PDE)‐4 inhibitor

Apremilast has been used at standard dose (with standard initial dose escalation) in studies on patients with PPP: A multicenter single‐arm study in Germany on 20 patients achieved a PPPASI‐50 response in 61.9% of the patients and a median PPPASI reduction by 57.1% after 20 weeks of treatment.^103^ A Japanese placebo‐controlled study with 90 participants confirmed these promising results and also showed a rapid reduction in the number of pustules (Table 4). At week 16, the mean PPPASI reduction was 64.3% in the apremilast group compared to 42.4% in the placebo arm. Moreover, almost 40 patients with predominantly positive therapeutic response have been described in case series and case reports.^105^, ^106^, ^107^, ^108^, ^109^

### Janus kinase (JAK) inhibitors

There are several promising case reports and case series on JAK inhibitors, in particular with upadacitinib, tofacitinib, and baricitinib.^110^, ^111^, ^112^, ^113^, ^114^, ^115^, ^116^ In a German case series with five patients predominantly refractory to treatment, a rapid response within the first four weeks was observed.^117^ However, the EMA recommendations stipulate the use of JAK inhibitors for treatment of patients aged 65 years or older, patients with increased risk of severe cardiovascular events (such as myocardial infarction or stroke), smokers or patients who have smoked for a longer period in the past, and patients with an increased risk of cancer, but only if no appropriate alternative treatments exist. JAK‐inhibitors should be used with caution in patients with risk factors for blood clots in the lung and deep veins (venous thromboembolism, VTE) exceeding those mentioned above. Moreover, doses should be reduced in patient groups at an increased risk of VTE, cancer, or severe cardiovascular events, if possible.^118^

### Biologic therapy

In recent years, significant progress in treating PPP has been made using inhibitors of TNF, IL‐23, and IL‐17, although the success rates are not as high as in PV.

Among biologics, IL‐23 and IL‐17RA inhibitors, in particular, have shown good efficacy in treatment of PPP. In contrast, studies on other cytokine inhibitors, such as ustekinumab, spesolimab, or IL‐1 inhibitors, have provided inconsistent or largely negative results.

### TNF inhibitors

The available data on TNF inhibitors in PPP are limited, possibly due to the risk of paradoxical reactions with pustular palmoplantar lesions during their use in other indications.^32^ A small Canadian placebo‐controlled study with etanercept showed a significant PPPASI reduction after 24 weeks compared to baseline in the group receiving only etanercept. No benefit was observed after twelve weeks of treatment with etanercept – again compared to placebo (Table 4).^119^

### Ustekinumab (IL12/23 inhibition)

Ustekinumab is often used if paradoxical reactions occur with TNF inhibitors.^120^ The studies performed in relation to PPP are inconsistent. A placebo‐controlled study showed no benefit while an open study conducted in the USA found an improvement in the majority of patients. Case reports emphasize the varying observations, which may be attributed to therapy duration and dosage.^38^, ^63^, ^121^

### IL‐23p19 inhibitors

Among IL‐23 inhibitors, the best evidence is available for guselkumab (Table 4), which was approved for the therapy of PPP in Japan in 2018,^122^, ^123^ followed by risankizumab.^124^

For guselkumab, three randomized, placebo‐controlled trials conducted in Japan demonstrate a significant and sustained improvement of PPPASI scores and quality of life until week 52. A German open, multicenter study on 50 patients with PPP confirmed these results without identifying differences in the response with respect to disease duration, smoking status, body weight, gender, or concomitant psoriasis (Table 4).^125^, ^126^, ^127^

A Japanese study on risankizumab in PPP was published recently. 41% of the patients treated with risankizumab (n = 61) showed a PPPASI‐50 response at week 16 compared to 24.1% of the patients treated with placebo (n = 58). This significant effect was not achieved for the PPPASI‐75 response.^128^ In addition, retrospective analysis of 16 patients treated with IL‐23 inhibitors (guselkumab, tildrakizumab, risankizumab) demonstrated positive results with PPPASI‐50 and PPPASI‐75 responses.^129^

### IL‐17 AND IL‐17RA INHIBITORS

#### Secukinumab (IL‐17A antagonist)

The 52‐week RCT 2PRECISE on secukinumab showed no superiority over placebo after 16 weeks. A marked improvement under secukinumab therapy was seen after 52 weeks. Further improvement of PPP was achieved in the extension study over 148 weeks.^130^, ^131^ A retrospective Spanish cohort study showed a PPPASI‐75 response in more than 50% of patients after two years of treatment (Table 4).^132^

#### Brodalumab (IL‐17 receptor A (IL‐17RA) antagonist)

A placebo‐controlled phase III trial (RCT) on 126 patients with PPP showed significant superiority of brodalumab over placebo with respect to PPPASI‐50 and PPPASI‐75 responses after 16 weeks. The results of the open 52‐week phase have not yet been published^133^ (Table 4).

#### Other IL‐17 inhibitors

In a retrospective analysis on 21 patients with PPP conducted in France, bimekizumab, a specific inhibitor of IL‐17A and IL‐17F, resulted in complete clearance in 17 patients after 1–4 months of treatment.^134^ A prospective placebo‐controlled study with bimekizumab (NCT07219420) in PPP will start in the near future (https://clinicaltrials.gov/search?cond=palmoplantar%20pustulosis&intr=Bimekizumab). Although a Chinese study with the IL‐17A antagonist ixekizumab showed a rapid response with intralesional administration, the method is considered impracticable.^135^

### Spesolimab (IL‐36 receptor antagonist)

In a European placebo‐controlled phase IIa study, spesolimab, a human monoclonal antibody against IL‐36R, did not meet the primary endpoint defined as achievement of PPPASI‐50 at week 16,^136^ (Table 4).

### IL‐1 inhibitors

In a placebo‐controlled study, the IL‐1 receptor antagonist anakinra showed no superiority over placebo in PPP. Moreover, treatment was discontinued in two case reports due to lack of efficacy,^137^, ^138^ (Table 4).

### Dupilumab

Dupilumab, a monoclonal antibody against the alpha subunit of the receptors of IL‐4 and IL‐13, is approved for the treatment of moderate to severe atopic dermatitis and prurigo nodularis. In a prospective study with dupilumab conducted in China on patients with PPP without psoriasis in personal or family history, nine of ten patients achieved a PPPASI‐75 response after 16 weeks.^139^

New promising therapeutic approaches have emerged form a case series on dupilumab.^139^ Moreover, future studies on JAK and TYK2 inhibitors might provide additional progress, given that individual successful treatment cases and case series have been published already.

### Further studies and registries

Further studies on treatment of PPP are required. According to the information from *ClincalTrials.gov*, a prospective study on deucravacitinib, a Tyk2 inhibitor, is currently recruiting in the USA (NCT05710185), and a study in China (NCT07000630) is planned.^140^

First studies on topical JAK inhibitors, for example, the pan‐JAK inhibitor delgocitinib, are in preparation (NCT07013201). The IL‐36 receptor antagonist imsidolimab is in clinical development (NCT03633396).^141^ Regarding sonelokimab, a bispecific nanobody inhibiting IL‐17A and IL‐17F, a placebo‐controlled phase II study on PPP is being conducted in Germany (https://euclinicaltrials.eu/ctis‐public/view/2024‐513305‐32‐00?lang=en). In the future, a Japanese registry (https://clinicaltrials.gov/study/NCT04459507) will provide additional information on this complex disease, similar to the German registry ppBest (https://www.ppbest.de).

Apart from the pharmacological therapy, optimizing the lifestyle is mandatory for treatment success.

## MANAGEMENT OF PPP

During management of PPP, trigger factors should be recognized and avoided or treated,^142^ (see checklist, Table 5). If applicable, patients should be informed about the importance of non‐smoking and nicotine abstinence, given that the positive effects of non‐smoking on the course of PPP have been confirmed in studies.^143^ Being overweight is also a risk factor of PPP, and active weight management should therefore be considered, especially if there is no response to the recommended therapies. Comorbidities must be taken into account when selecting the systemic therapy, and an interdisciplinary management is recommended in these cases.

**TABLE 5:** Checklist: Management of risk and/or trigger factors of palmoplantar pustulosis (PPP). The checklist provides a structured overview of key recommendations for managing PPP. It systematically addresses potential trigger factors, occupational aggravation, and relevant comorbidities, offering practical measures for clinical use.

Recommendations		Measures (examples)
**1. Determination of potential trigger factors** ∙ Smoking tobacco	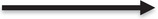	Nicotine abstinence (smoking cessation program)
∙ Overweigh	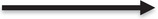	Weight reduction (dietary counseling, sports activity)
∙ Infectious foci	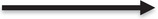	Treatment according to specialists (e. g., ENT, dentists)
∙ Mechanical irritation	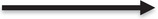	Avoid as much as possible
∙ Psychological stress	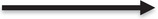	Stress reduction (psychological support)
∙ Drugs: Paradoxical PPP (e. g., caused by TNF inhibitors)	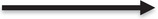	Consider switching
**2. Assessment of occupational aggravation**	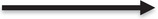	Notification to workers' compensation association (initiation dermatologist's report)
**3. Screening for comorbidity** Bone and joint involvement Psoriasis vulgaris Metabolic syndrome (diabetes, arterial hypertension, hyperlipidemia, overweight) Psychiatric disorders		

## SUMMARY ON THE THERAPY OF PPP

**FIGURE 5:**
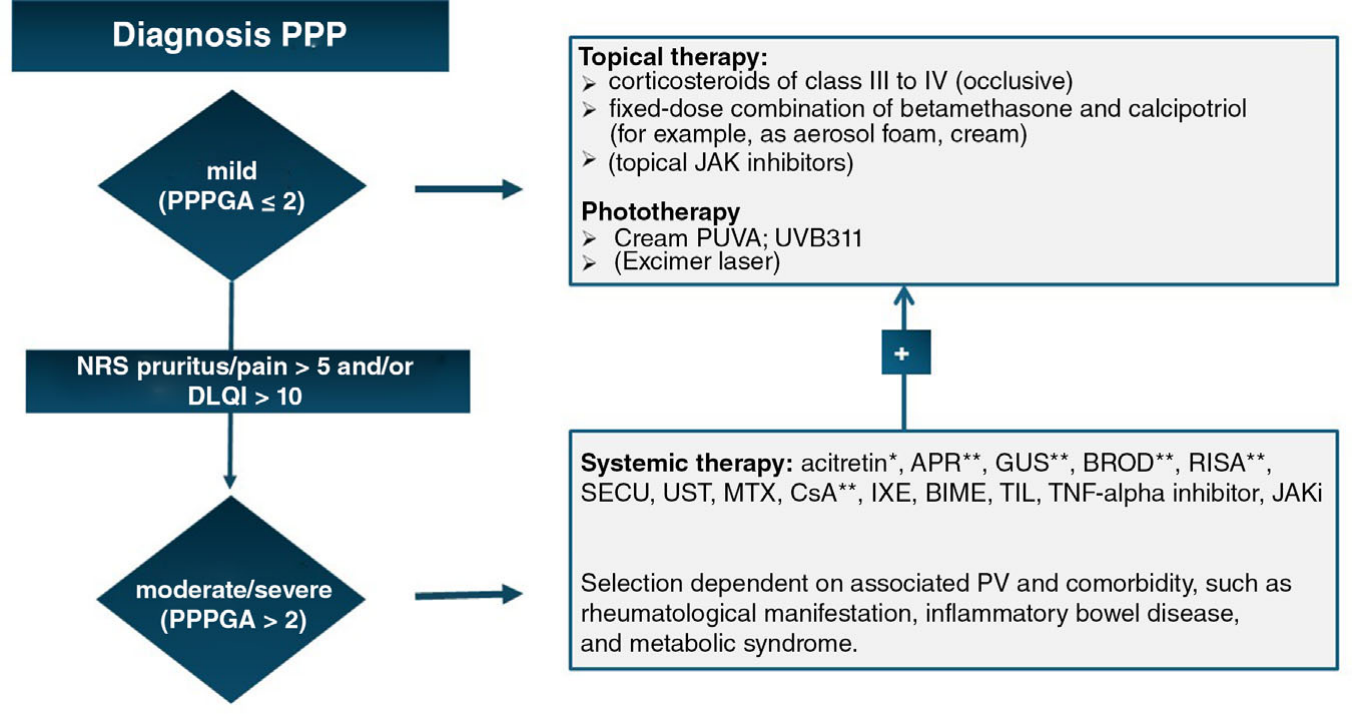
Treatment algorithm for palmoplantar pustulosis (PPP). The algorithm presents a simplified scheme for therapeutic management of PPP depending on severity and any comorbidities. If concomitant bone/joint involvement or plaque psoriasis is present, systemic therapy is guided by the extent of joint and skin involvement as well as existing comorbidities. Treatment is also based on the severity of PPP. For moderate to severe cases, as well as mild cases with a Dermatology Life Quality Index (DLQI) > 10 and/or Numeric Rating Scale (NRS) > 5, systemic therapy is indicated. *Approved therapy for PPP, also in absence of plaque psoriasis; **Randomized controlled trials (RCTs) with achievement of the primary endpoint available for PPP. Bold systemic therapies represent preferred options due to highest evidence. *Abbr*.: Psoralen + UVA phototherapy; UVB311 = UVB phototherapy with 311 nm; PV = Psoriasis vulgaris/Plaque Psoriasis; ADA = Adalimumab; APR = Apremilast; BIME = Bimekizumab; BROD = Brodalumab; CsA = Ciclosporin A; GUS = Guselkumab; IXE = Ixekizumab; MTX = Methotrexate; RISA = Risankizumab; SECU = Secukinumab; TIL = Tildrakizumab; TNF = Tumor Necrosis Factor; UST = Ustekinumab.

The therapy of PPP continues to pose a major challenge. Topical ultrapotent steroids under occlusion are part of the standard therapy, either alone or supplementary to phototherapy and/or systemic therapy (Figure 5). Topical PUVA photochemotherapy, in particular, has become an established part of PPP treatment. UV phototherapies are time‐consuming for those affected and not suitable for long‐term treatment. Acitretin is the only drug approved for systemic therapy of PPP. Often, however, it cannot be used or must be discontinued due to the slow onset of action, the unfavorable side effect profile in patients with metabolic syndrome, and the obligate teratogenicity. While ciclosporin shows a faster and more effective onset of action, it is not a good option for long‐term therapy. MTX was not convincing in the described analyses on PPP. In contrast, apremilast showed convincing results in a Japanese and a German prospective study. It is particularly effective in reducing pustules. Other *small molecules* like JAK inhibitors have been shown to be effective in initial case reports and case series. Some biologics, such as IL‐23 or IL‐17RA inhibitors, have shown good response rates (Table 4). The IL‐23 inhibitor guselkumab has been approved in Japan. With respect to patient management on guselkumab, it is important to note that the data on PPPASI improvement at the early measurement times are outperformed by the data after 1 or 2 years. The IL‐36 receptor antagonist spesolimab or the IL‐1 inhibitor anakinra failed to live up to expectations in PPP. Further studies are required to evaluate the efficacy profile of biologics with other targets, such as IL‐17A/F.

## CONFLICT OF INTEREST STATEMENT

The following authors acted as advisors and/or received honoraria or reimbursement of travel expenses for speakers and/or received grants and/or participated in clinical studies of the following companies:

Tanja Fetter: Biogen, AstraZeneca, Incyte, ADF

Dagmar Wilsmann‐Theis was advisor, speaker or principal investigator for studies of AbbVie, Almirall, Amgen, Biogen, Boehringer Ingelheim, Bristol Myers Squibb, Celgene, GlaxoSmithKline, Hexal, Incyte, Janssen‐Cilag, Leo Pharma, Eli Lilly, Medac, Merck Sharp & Dohme Corp., Moonlake, Novartis, Pfizer, and UCB Pharma.

Ulrich Mrowietz received honoraria as advisor and/or speaker for the following companies: AbbVie, Aditxt, Almirall, Amgen, Biogen, Boehringer‐Ingelheim, Bristol‐Myers Squibb, Eli Lilly, Immunic, Janssen‐Cilag, LEO Pharma, Merck, Sharp & Dohme, Novartis, Phi‐Stone, SelectION, UCB Pharma, UNION therapeutics.

Rotraut Mössner received honoraria as advisor and/or speaker and/or received research grants and/or participated in clinical studies of the following companies: AbbVie, Amgen, Almirall, Biogen IDEC, Böhringer‐Ingelheim, Celgene, Janssen‐Cilag, Leo Pharma, Lilly, Moonlake, MSD SHARP & DOHME, Novartis Pharma, Pfizer, and UCB.

Robert Sabat received research grants or contracts for clinical studies from AbbVie, Boehringer Ingelheim Pharma, Celgene/Amgen, Celgene/Bristol Myers Squibb, Charité Research Organization, CSL Behring, ICON, IQVIA RDS, Incyte, Janssen‐Cilag/Janssen Research & Development, MoonLake Immunotherapeutics, Novartis, Parexel, Rheinische Friedrich Wilhelms University Bonn, Sanofi Aventis, TFS, and UCB Biopharma, which were paid to his organization; honoraria for lectures, advisory services, or participation in advisory boards of AbbVie, Almirall Hermal, Amgen, Bayer Schering Pharma, Bruno Bloch Stiftung, Janssen‐Cilag/ Janssen Research & Development, Novartis, UCB Biopharma, University Medicine Greifswald and Wound Network Berlin‐Brandenburg; moreover, he is member of the International Psoriasis Council (unpaid) and speaker of the Psoriasis Working Group of the Dermatological Research Working Group (ADF) (unpaid).

All other authors declare that the research was conducted in absence of any commercial or financial interests and relationships that might be interpreted as potential conflict of interest.

## FUNDING

Tanja Fetter received a grant from the BONFOR program of the University of Bonn (Gerok position, Funding Code 2023‐1A‐17). The other authors declare that they did not receive any support for the research, authorship, and/or publication of this article.

## [CME Questions/ Lernerfolgskontrolle]

1. Welche Kurzbeschreibung trifft auf die palmoplantare Pustulose (PPP) zu?
  - Sie ist eine akute, selbstlimitierende Erkrankung
  - Sie ist genetisch identisch mit der Psoriasis vulgaris
  - Sie manifestiert sich mit sterilen Pusteln an Handflächen und Fußsohlen
  - Männer und Frauen sind etwa gleichhäufig betroffen
  - Es gibt aktuelle Leitlinien für die Therapie
2. Welche der folgenden Erkrankungen sollte bei der Diagnose einer palmoplantaren Pustulose (PPP) differenzialdiagnostisch in erster Linie in Betracht gezogen und ggf. ausgeschlossen werden?
  - Vitiligo
  - Tinea manus/pedis
  - Lupus erythematodes
  - Rosacea
  - Erythema exsudativum multiforme
3. Welcher der folgenden Faktoren gilt als der wichtigste Provokationsfaktor für die palmoplantare Pustulose (PPP)?
  - Warme Bäder
  - Mangel an Vitamin D
  - Regelmäßiger Konsum von Alkohol
  - Tabakrauchen
  - UV‐Exposition
4. Eine „paradoxe PPP” tritt am häufigsten in Zusammenhang mit der Behandlung welcher Medikamentenklasse auf?
  - Retinoide
  - Ciclosporin
  - TNF‐α Blocker
  - Fumarsäureester
  - Topische Kortikosteroide
5. Welche Aussage zu Knochen/Gelenkbeteiligung bei der PPP trifft zu?
  - Eine PPP ist ein notwendiges Kriterium für das Vorliegen einer pustulösen Arthroosteitis
  - Eine PPP ist ein notwendiges Kriterium für das Vorliegen eines SAPHO‐Syndroms
  - Patienten mit PPP mit einer nichtinfektiösen Arthritis mehrerer Fingergelenke erfüllen immer die CASPAR‐Kriterien für eine Psoriasisarthritis
  - Bei Patienten mit PPP liegt häufig ein positiver Rheumafaktor vor
  - Eine sternokostoklavikulärer Hyperostose ist ***keine*** typische Manifestation der Knochen/Gelenkbeteiligung bei der PPP
6. Welche Aussage trifft auf die genetische Basis der PPP zu?
  - Sie ist eng mit dem PSORS1‐Locus assoziiert
  - Sie unterscheidet sich genetisch deutlich von der Psoriasis vulgaris, insbesondere fehlt die PSORS1‐Assoziation
  - Sie ist genetisch überwiegend durch Mutationen im *IL36RN*‐Gen bedingt
  - Sie ist durch eine autosomal‐dominante Vererbung gekennzeichnet
  - Die Genetik der PPP ist vollständig aufgeklärt und zeigt keine Unterschiede zur GPP
7. Welche Immunmediatoren spielen eine wichtige Rolle in der Pathogenese der palmoplantaren Pustulose (PPP), die durch aktivierte Keratinozyten sezerniert werden und neutrophile Granulozyten anlocken?
  - Interleukin‐4 (IL‐4) und IL‐13
  - Chemokine CXCL6 und CXCL8
  - Interferon‐gamma (IFN‐γ) und IFN‐α
  - Interleukin‐10 (IL‐10) und IL‐31
  - Chemokine CXCL9 und CXCL11
8. Welche Aussage trifft am besten auf die Pathogenese der PPP zu?
  - Sie beginnt im Bereich der Haarfollikel mit T‐Zell‐Aktivierung
  - Sie beginnt um das Acrosyringium, dem intraepidermalen Ausgangskanal der Schweißdrüsen, mit neutrophiler Infiltration
  - Sie ist eine direkte Folge einer Pilzinfektion der Haut
  - Sie entsteht durch eine autoimmune Reaktion gegen Keratinozyten
  - Die Pautrier‐ Mikroabszesse sind typisch für die PPP
9. Welche Aussage beschreibt die Wirksamkeit der UV‐Therapie bei PPP am besten?
  - UV‐B ist effektiver als PUVA bei der Behandlung der PPP
  - Die Bade PUVA‐Therapie ist zur Therapie der PPP zugelassen
  - UV‐Therapie ist bei PPP kontraindiziert
  - UV‐Therapie ist nur bei Psoriasis vulgaris wirksam, nicht bei PPP
  - Excimer‐Therapie darf bei der PPP nicht eingesetzt werden
10. Welche systemische Therapie ist in Deutschland für die Behandlung der PPP zugelassen?
  - Methotrexat
  - Ciclosporin
  - Acitretin
  - Apremilast
  - Guselkumab

Liebe Leserinnen und Leser, der Einsendeschluss an die DDA für diese Ausgabe ist der 30. Juni 2026.

Die richtige Lösung zum Thema Einsatz von nichtinvasiver optischer Bildgebung in der Dermatologie in Heft 11/2025 ist: 1b, 2c, 3a, 4a, 5c, 6b, 7c, 8c, 9c, 10b

Bitte verwenden Sie für Ihre Einsendung das aktuelle Formblatt auf der folgenden Seite oder aber geben Sie Ihre Lösung online unter http://jddg.akademie-dda.de ein.
